# An estriol-eluting pessary to treat pelvic organ prolapse

**DOI:** 10.1038/s41598-022-23791-9

**Published:** 2022-11-21

**Authors:** Jingjunjiao Long, Ghada Zidan, Ali Seyfoddin, Stephen Tong, Fiona C. Brownfoot, Prathima Chowdary

**Affiliations:** 1 Drug Delivery Research Group, Faculty of Health and Environmental Sciences, School of Science, Auckland University of Technology, Auckland, New Zealand; 2 Translational Obstetrics Group, Department of Obstetrics and Gynaecology, University of Melbourne, Mercy Hospital for Women, 163 Studley Road, Heidelberg, VIC 3084 Australia; 3 Obstetric Diagnostics and Therapeutics Group, Department of Obstetrics and Gynaecology, University of Melbourne, Mercy Hospital for Women, 163 Studley Road, Heidelberg, VIC 3084 Australia; 4 Department of Obstetrics and Gynaecology, University of Auckland, Auckland, New Zealand; 5 Department of Orthopedics, Orthopedic Research Institute, West China Hospital, Sichuan University, Chengdu, 610041 China

**Keywords:** Medical research, Preclinical research, Health services, Public health, Quality of life, Therapeutics

## Abstract

Pelvic organ prolapse affects up to 50% of parous women. Commonly used treatment options have unwelcome attributes; pessaries can cause erosion and estrogen creams need to be applied frequently, which is inconvenient and difficult to administer. This study involved the development of an estriol-releasing pessary utilising 3D printing molds. We incorporated varying amounts of estriol (1%, 10% and 15%) into the silicone pessary. We optimised the mechanical aspects of the pessary so it had a similar strength to commercially available pessaries. We investigated estriol release from the pessary over 3 months. We explored possible interactions between the drug and polymers via FTIR. The MED-4870 silicone ring with similar mechanical strength to pessaries currently used to treat pelvic organ prolapse. The medical pessaries present a sustained release in simulated vaginal fluid over 3 months. The pessary with 10% estriol delivered the optimal dose at 0.8 mg each week. Mechanical strength of this pessary showed no difference after emersion in simulated vaginal fluid for 3-month, supporting the long-term application. An estriol-loaded pessary was successfully developed to treat pelvic organ prolapse with sustained release of estriol over 3 months. This pessary provides promising potential to treat pelvic organ prolapse and vaginal atrophy.

## Introduction

Pelvic organ prolapse is a common condition affecting up to 50% of parous women which can cause local discomfort, voiding, defecatory and sexual dysfunction and adversely impact quality of life^1^. Management of symptomatic pelvic organ prolapse usually involves pelvic floor physical therapy and the use of pessaries^2,3^. Complications associated with pessaries are common and include discomfort, vaginal erosion, bleeding, and fecal impaction^4–7^. These side effects are often exacerbated in the presence of poorly fitted devices and vaginal atrophy with a short vaginal length and a wide vaginal introitus^8–10^.

The size of pessaries is a key parameter to enable proper fitting. Wu, et al.^11^ had performed an initial pessary fitting to find a proper size where (1) the pessary was not expelled, (2) the patient could not feel the pessary, and (3) the pessary did not descend to introitus during testing. In this way, 81 patients (74%) were successfully fitted with pessaries while 29 (26%) were not able to use them. A generic pessary size is 55–100 mm and will fit the majority of women. For patients with a short vaginal length or wide introitus, a customized pessary could be made using 3D printing. Selective Laser Sintering (SLS) 3D printing allows devices to be customised in a fraction of the time and cost compared to traditional mold design^12^. This allows us to rapidly prototype and individualise devices to meet patient’s needs^13^.

To reduce vaginal erosion associated with atrophy, estrogen creams or E-string are often prescribed^14–17^. These creams need to be applied frequently and can be difficult to administer. Some women may be unsuitable for treatment with ESTRING vaginal delivery system, in particular those with short narrow vaginas due to previous surgery, or the effects of vaginal atrophy, or those with a degree of uterovaginal prolapse severe enough to prevent retention of the ring. This challenge, alongside the problems with ill-fitting pessaries, could be alleviated by using appropriately individualised, medicated pessaries with slow-release estriol.

Similar modelling of combining silicon with release of hormone is widely used clinically in the form of a Mirena (levornogestrol intrauterine device). Mirena’s active ingredient, levonoruigestrel (LNG), is dispersed in a silicone (polydimethylsiloxane) reservoir on the stem. This reservoir contains 52 mg of LNG, and is covered by a polydimethylsiloxane membrane which allows for a controlled release of the hormone over time^18^.

We embarked on a study to see whether we could 3D print an estriol eluting pessary. The overall aim of the study was to develop and evaluate a silicone pessary that could release estriol at a clinically relevant dose (0.5 mg every week) over three months. The specific objectives of this study were: (1) to investigate the feasibility of fabricating silicone pessary rings using 3D printed molds; (2) to incorporate various amounts of estriol into two different silicone matrices (MED-4830 and MED-4870) and (3) to optimise the formulation by characterising the physical and chemical properties of this system and evaluating in vitro drug release over three months.

## Results

### Preliminary optimization experiments

**Figure 1:**
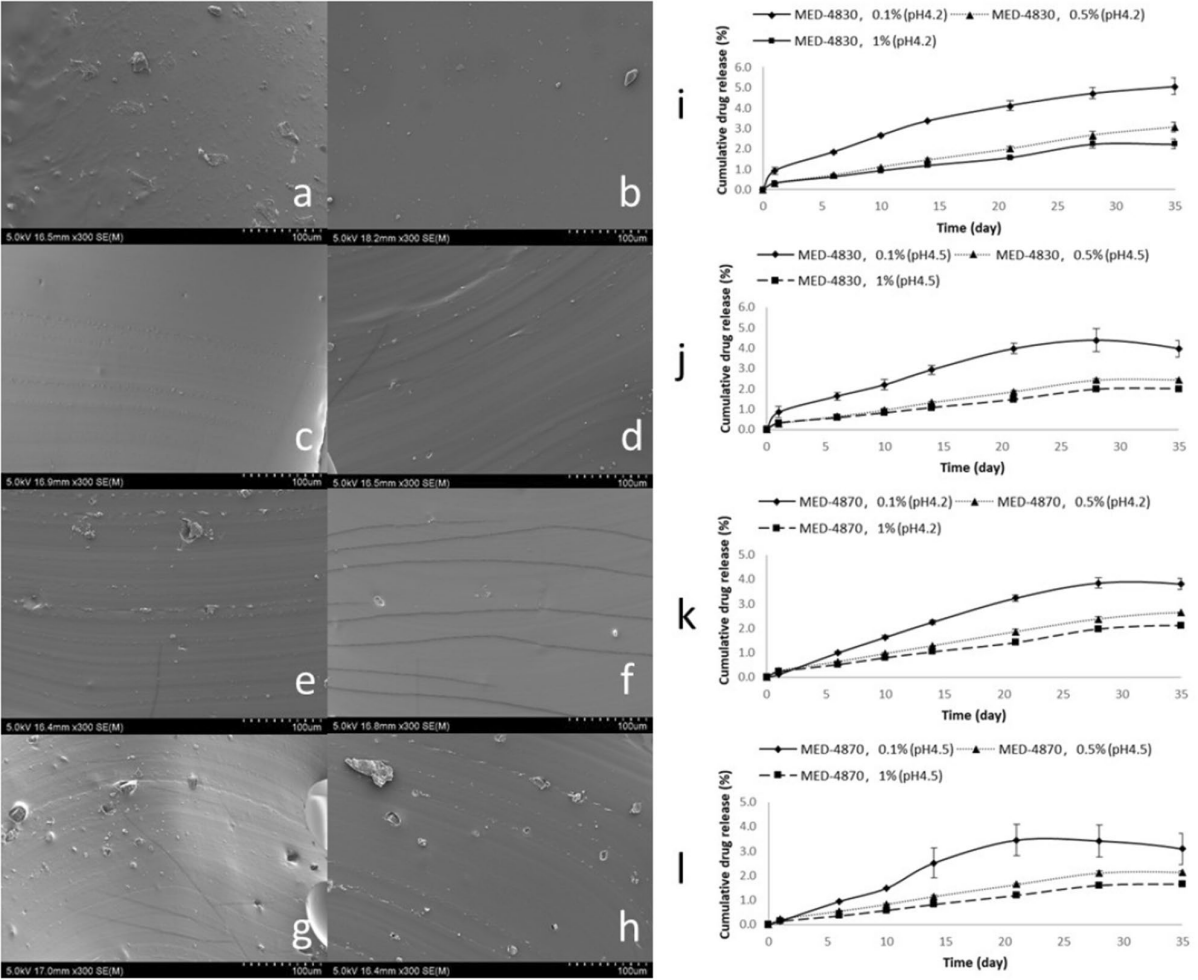
Scanning electron microscope cross section micrographs of MED-4830 and MED-4870 films(**a–h**): (**a**) MED-4830, drug-free sample; (**b**) MED-4870, drug-free sample; (**c**) MED-4830, 0.1% drug content; (**d**) MED-4870, 0.1% drug content; (**e**) MED-4830, 0.5% drug content; (**f**) MED-4870, 0.5% drug content; (**g**) MED-4830, 1% drug content; and (**h**) MED-4870, 1% drug content. In vitro release of estriol from silicone films based on different silicone materials, drug contents and release conditions (**i–l**): (**i**) MED-4830 silicone films released in simulated vaginal fluid (SVF) of pH 4.2; (**j**) MED-4830 silicone films released in SVF of pH 4.5; (**k**) MED-4870 silicone films released in SVF of pH 4.2; and (**l**) MED-4870 silicone films released in SVF of pH 4.5; solid line represents 0.1% drug content, dotted line represents 0.5% drug content and dashed line represents 1% drug content.

In order to explore the feasibility of encapsulating estriol into silicone carriers and the controllability of release behaviours, we studied in vitro release of the films. We did this by comparing the difference between silicone materials, drug contents and release conditions in different pH solutions used to simulate premenopausal and postmenopausal fluids. We found MED-4830 and MED-4870 films presented similar results in release profiles under both normal vaginal pH (4.2) and post-menopausal vaginal pH (4.5) (Fig. 1i-l). However, increased drug loading possibly delivers longer and more concentrated estriol than that with reduced drug loading. This is possibly due to more micropores and micro-channels created by the drug crystals in the high drug-loading group that is observed by scanning electron microscopy (Fig. 1a-h), promoting higher media communication and faster drug diffusion. Three silicone films with varying amount of estriol were tested for drug release as preliminary work before manufacturing the rings. An average was calculated and over a period of 35 days, 5.1, 3.1 and 2.2% of estriol, equal to 0.05, 0.15 and 0.22 mg respectively, were released from three silicone films.

### 3D printing and incorporation of estriol into the silicone rings

**Figure 2:**
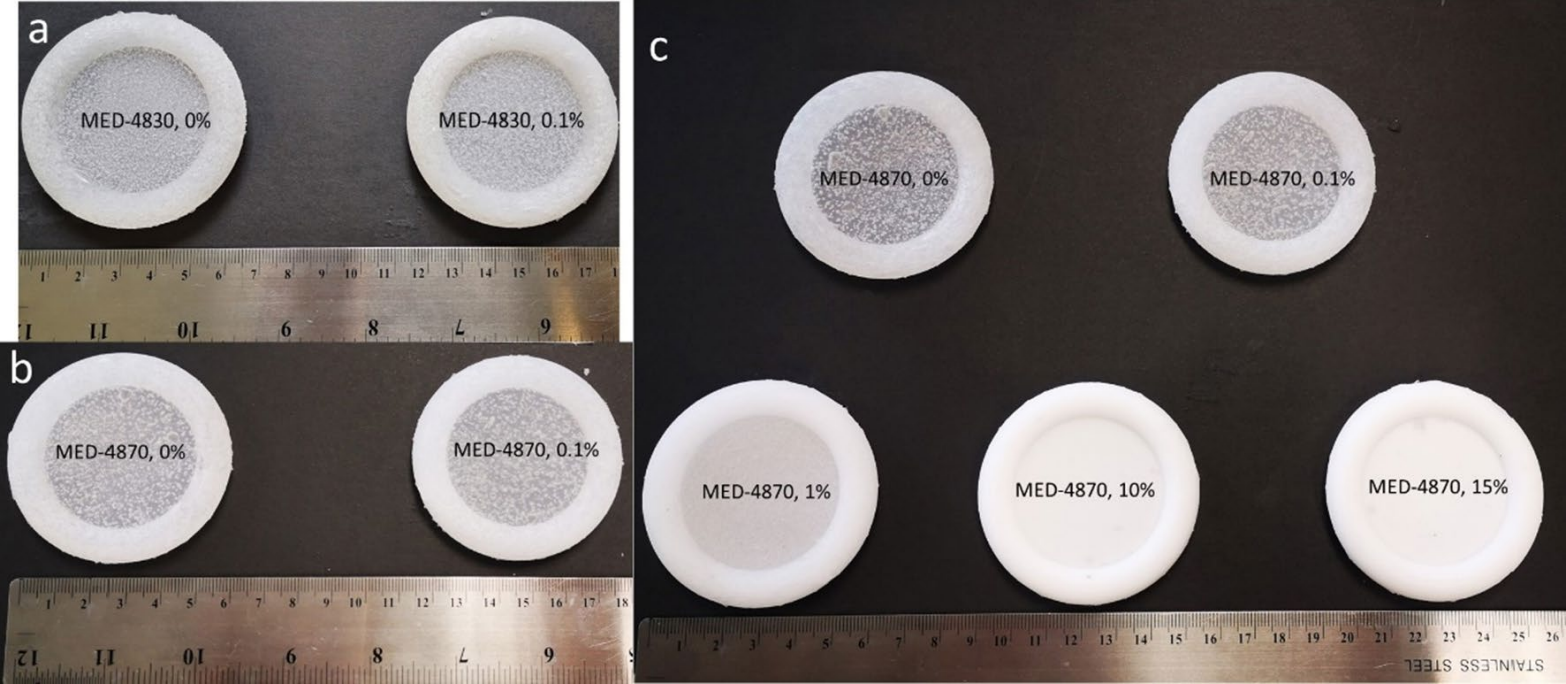
Manufacture trial (**a**) and (**b**) and improved trial (**c**) of silicone rings with different drug contents.

We made films with silicone and estriol to determine whether they can be combined. All samples presented smooth surfaces and cross-section indicating the silicone was properly cured with uniform texture. The two different grades of silicone, MED-4830 and MED-4870 materials, were used as these medical-grade silicone elastomers are the industry standard for pessary manufacture capable of providing sufficient mechanical strength^19^. Both MED-4830 and MED-4870, with/without estriol, showed solidification after curing and the predefined shape was well retained as fully toroidal (Fig. 2). All rings almost matched the original design mode which is a commercially bought Milex pessary ring with outer diameter value of 64 mm, membrane diameter value of 44 mm and cross-sectional diameter value of 10 mm (Table 1). The fabrication accuracy (which was measured as experimental dimension/designed dimension of OD, MD and CSD) was all above 99%, indicating the high feasibility of consistently fabricating customised pessary rings through 3D printed molds. MED-4870 rings were noticeably stronger with higher mechanical strength (confirmed in compression tests in the following section), which is similar to the commercial pessary ring. Therefore, MED-4870 silicone elastomer was chosen for optimizing pessary rings.

**Table 1:** Morphological dimensions and weight of silicone pessary rings.

Silicone and estriol loading dose	Weight (g)	OD (mm)*	MD (mm)**	CSD (mm)***
MED-4830 ring with no estriol	16.15 ± 0.22	63.49 ± 0.14	43.67 ± 0.18	10.08 ± 0.03
MED-4830 ring with 0.1% estriol loading	16.57 ± 0.50	63.32 ± 0.15	43.81 ± 0.18	10.19 ± 0.09
MED-4870 ring with no estriol	18.04 ± 0.62	63.63 ± 0.11	43.87 ± 0.09	10.18 ± 0.10
MED-4870 ring with 0.1% estriol loading	18.21 ± 0.62	63.32 ± 0.15	43.81 ± 0.18	10.19 ± 0.09
MED-4870 ring with 1% estriol loading	17.74 ± 0.70	63.31 ± 0.08	43.68 ± 0.17	10.39 ± 0.18
MED-4870 ring with 10% estriol loading	19.22 ± 0.14	63.46 ± 0.09	43.70 ± 0.13	10.44 ± 0.10
MED-4870 ring with 15% estriol loading	18.10 ± 0.53	63.59 ± 0.03	43.62 ± 0.12	10.44 ± 0.06

*Outer diameter (OD), **Membrane diameter (MD), ***Cross-sectional diameter (CSD).

**Figure 3:**
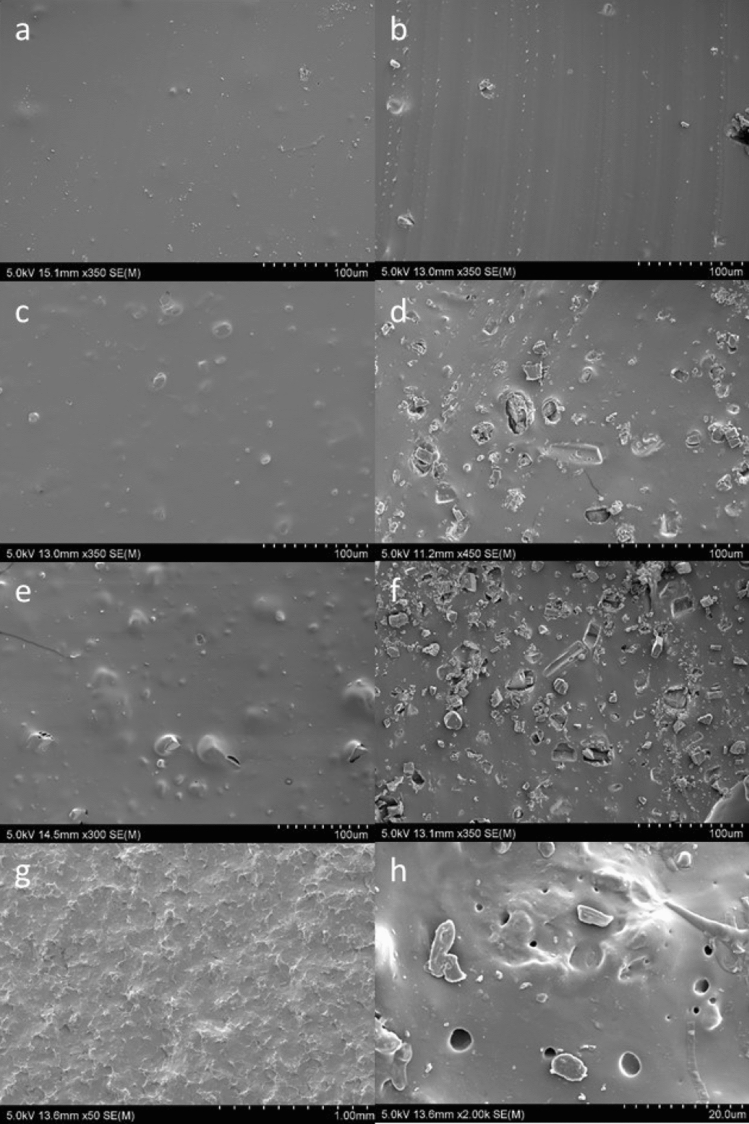
SEM micrographs of MED-4870 rings: (**a**) surface scan of MED-4870, 1% drug content sample; (**b**) cross-section scan of MED-4870, 1% drug content sample; (**c**) surface scan of MED-4870, 10% drug content sample; (**d**) cross-section scan of MED-4870, 10% drug content sample; (**e**) surface scan of MED-4870, 15% drug content sample; (**f**) cross-section scan of MED-4870, 15% drug content sample; (**g**) post submersion surface scan of MED-4870, 0.1% drug content sample after 4-month immersing in SVF solution; (**h**) post submersion cross-section scan of MED-4870, 0.1% drug content sample after 4-month immersing in SVF solution.

MED-4870 pessary rings were made with estriol drug content ranging from 0.1 to 15% (0.1, 0.5, 1, 10 and 15%). The surface and inner micro-structures of the improved formulations were studied by SEM. The surface and cross-sectional characteristics of MED-4870 rings, the MED-4870 ring with 1% estriol loading appeared smoother compared to MED-4870 ring with 10% estriol loading and MED-4870 ring with 15% estriol loading which had rough surfaces and more visible drug crystals (Fig. 3). This is likely due to the drug molecules themselves, which were being physically entrapped in the silicone matrix. (Fig. 3).

### Mechanical assessment

A successful ring pessary device must withhold sufficient pressure so it is not expelled during daily activities whilst retaining sufficient flexibility so it does not damage the vaginal tissue or cause discomfort to the patient^20^. Compression assessment is one of the most commonly practiced methods to evaluate the mechanical characteristics of pessary rings^20^.

**Figure 4:**
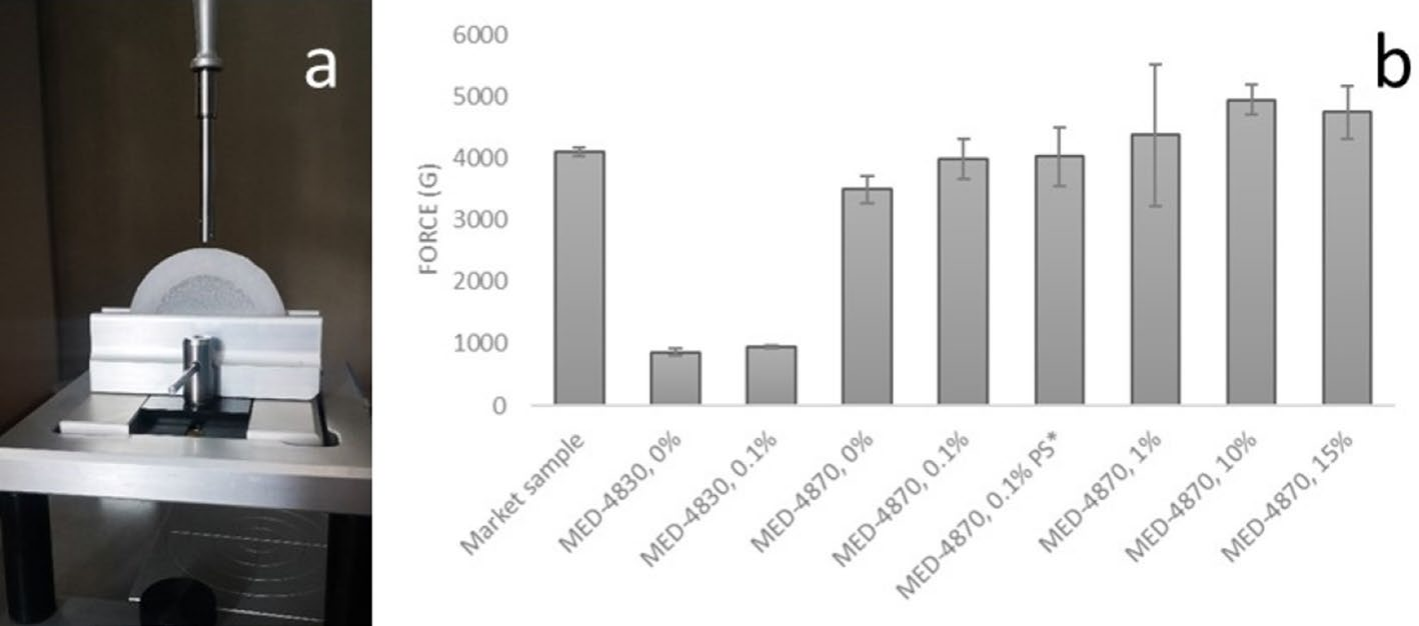
Compression test of silicone rings: (**a**) Compression probe and holder in texture analyser; (**b**) Compression forces in market sample and customised silicone rings (n $\ge$ 3 $\pm SD)$. PS* post submersion—treated with SVF (pH 4.2) for a period of 4-month at 35 ± 2 °C.

We performed compression testing and all samples returned to original toroidal shape without any visible deformation (Fig. 4). Pessary rings made of MED-4870 silicone exhibited much higher mechanical strength than that made of MED-4830 silicone. Specifically, MED-4870 ring with no estriol and MED-4870 ring with 0.1% estriol loading rings showed 3490 and 3986 g compression strength respectively whereas the compression strength for MED-4830 ring with no estriol and MED-4830 ring with 0.1% estriol loading rings was only 863 and 954 g respectively. All MED-4870 rings recorded similar mechanical strength (4946 $\pm$ 246 g) to the market non-drug eluting pessary sample (4112 $\pm$ 67 g) (Fig. 4b). Out of that, MED-4870 ring with 10% drug content showed highest compression force of 4946 $\pm$ 246 g. This is similar to the commercial pessaries. Consequently, MED-4870, 10% was suggested as the optimized formulation in accord with previous results.

Considering the long-term application of pessary rings, it is essential to inspect if the mechanical support of pessaries may change over time. Thus, a treatment of submerging MED-4870 ring with 0.1% estriol loading rings in simulated vaginal fluid pH 4.2) for a period of 4-month at 35 ± 2 °C was carried out and repeated with compression test on completion of submersion. Mechanical test also indicated no significant difference (p = 0.9304) on compression force between MED-4870 ring with 0.1% estriol loading before submersion (3986 $\pm$ 324 g) and MED-4870 ring with 0.1% estriol loading post submersion (PS) (4024 $\pm$ 474 g).

### Elemental analysis

**Figure 5:**
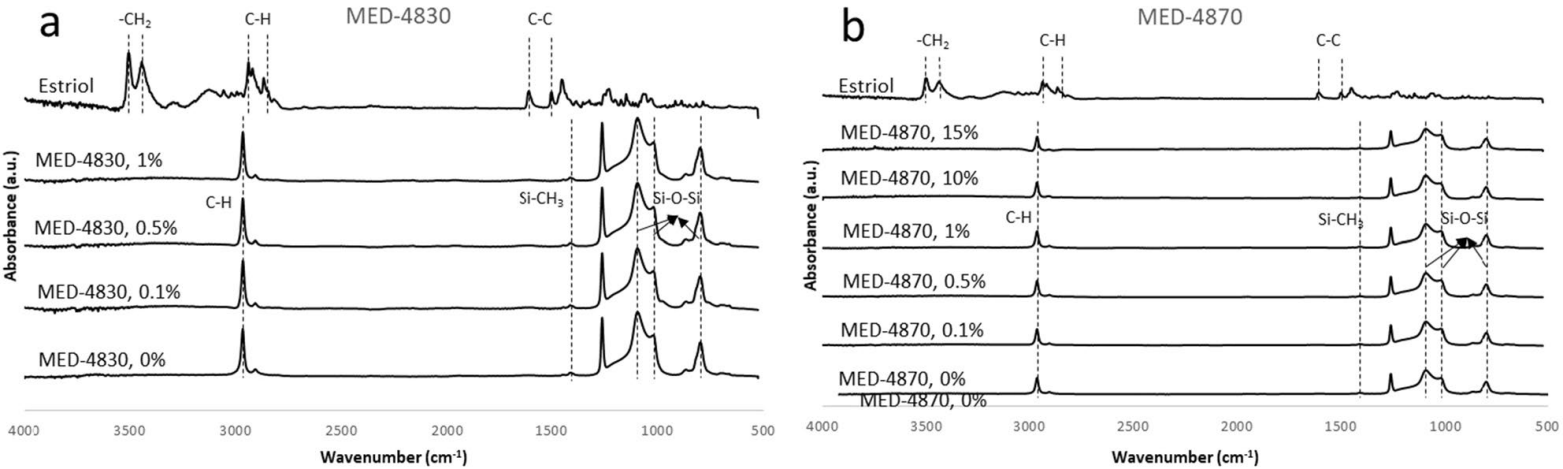
Fourier transform infrared (FTIR) spectra of MED-4830 (**a**) and MED-4870 (**b**) systems. MED-4870 ring with 0.1% estriol loading ring post submersion—treated with SVF (pH 4.2) for a period of 4-month at 35 ± 2 °C. The peaks of estriol and silicone were lined out and marked with chemical structures in figures. No clear peak of estriol in silicone samples was because the low ratio of estrio:silicone, the silicone mask the characteristic peaks of estriol.

Fourier-transform infrared (FTIR) spectroscopy is used to characterise the presence of specific chemical groups from silicones and drug, and also determine if any chemical interactions exist between the polymers and drug. Figure 5 shows the Fourier-transform infrared spectra of MED-4830 (a) and MED-4870 (b) silicone systems and their estriol-loaded composites.

The functional groups in each component (silicone and estriol) were detected as infrared spectrum of absorption and presented as specific peaks or bands (see Supplementary). After incorporating in the silicone matrix, the characteristic peaks of estriol were not detected in the initial estriol-silicone sample, possibly because the drug content was low. The majority polymer is expected to mask or dilute contributions of additives when the loading level is low^12^. We therefore performed further experiments with different drug loading doses and silicones to meet clinical need.

Considering the silicone material made up the large majority in pessaries, all MED-4870 silicone samples show similar spectra peaks. A small piece from each pessary was cut off and analysed. All the characteristic peaks of silicones were present in the estriol-loaded formulations without any noticeable peak shift or widening. This indicates no molecular interaction between the drug and the silicone carriers. Both estriol and silicone material preserve their chemical structures without any functional groups changing during the mixing and curing processes (Fig. 5).

### Estriol release from silicone rings

**Figure 6:**
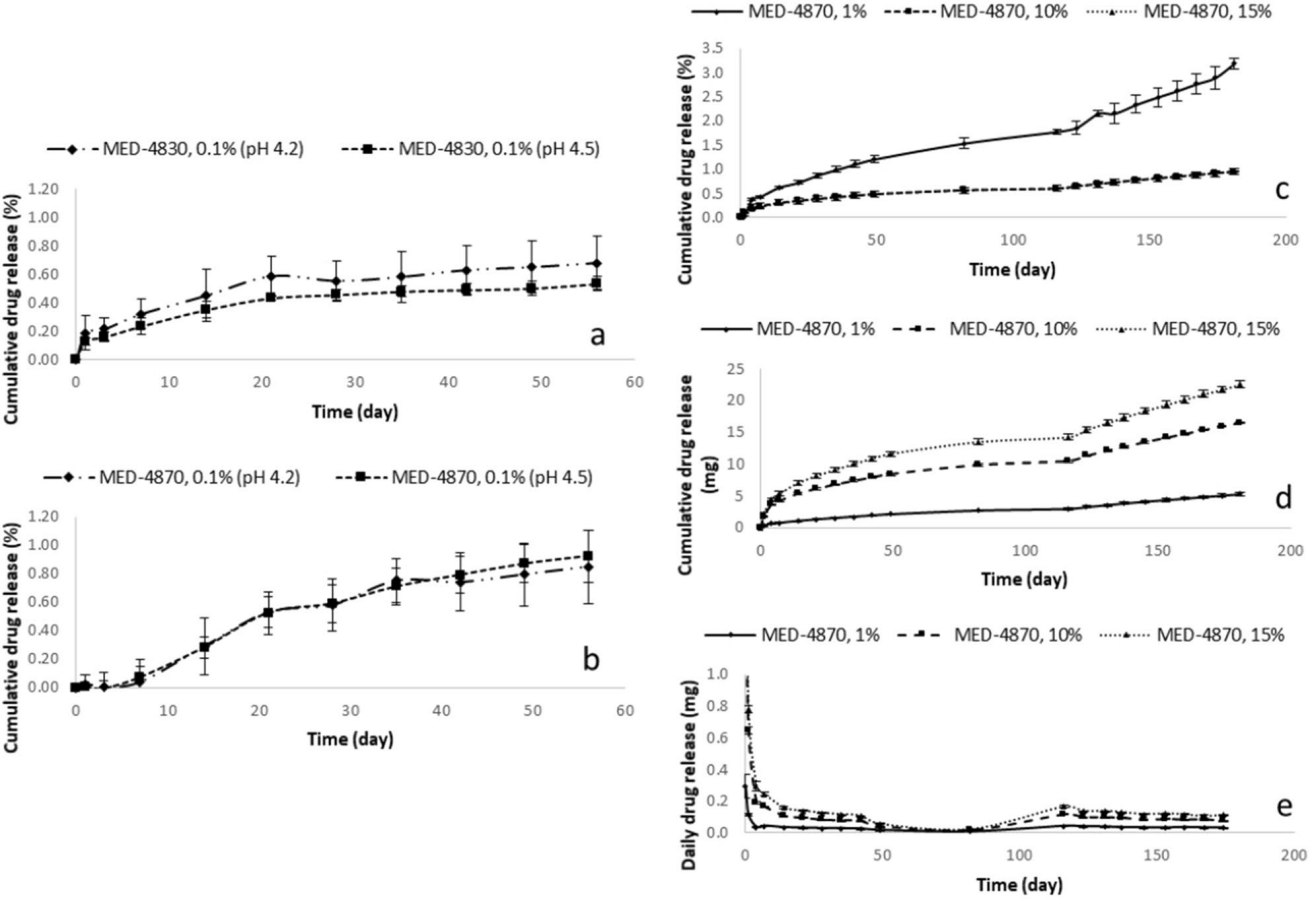
In vitro release of estriol from silicone rings: (**a**) Cumulative drug release in percentage (%): MED-4830 silicone rings with 0.1% drug content released in simulated vaginal fluid (SVF) of pH 4.2 and pH 4.5, (**b**) Cumulative drug release in percentage (%): MED-4870 silicone rings with 0.1% drug content released in SVF of pH 4.2 and pH 4.5; (**c**) Cumulative drug release in percentage (%): MED-4870 silicone rings with 1% drug content, 10% drug content and 15% drug content released in SVF of pH 4.5; (**d**) Cumulative drug release in amount (mg): MED-4870 silicone rings with 1% drug content, 10% drug content and 15% drug content released in SVF of pH 4.5; (**e**) Daily drug release in amount (mg): MED-4870 silicone rings with 1% drug content, 10% drug content and 15% drug content released in SVF of pH 4.5.

Considering post-menopause vaginal pH 4.5 is slightly higher than normal vaginal pH 4.2, the release of estriol from MED-4830 and MED-4870 rings were firstly studied in simulated vaginal fluid of both pH conditions. The 0.1%-estriol formulations exhibited a consistent release up to 56 days (Fig. 6a,b). Specifically, 0.68 and 0.53% of estriol (equal to 0.12 and 0.09 mg) were released from MED-4830 ring with 0.1% estriol loading in simulated vaginal fluid of pH 4.2 and 4.5; 0.85 and 0.93% of estriol (equal to 0.15 and 0.17 mg) were released from MED-4870 ring with 0.1% estriol loading under pH 4.2 and 4.5, respectively (n = 3, presented as means). The release performance of MED-4870 ring with 0.1% estriol loading rings presented a sustained and controllable delivery, however, the dose of released estriol (0.15–0.17 mg over 56 days) was way below clinical therapeutic concentration (Fig. 6b).

We increased the drug concentration in the MED-4870 rings ranging from 1 to 15%. All the samples were tested in the mechanical study and presented under mechanical stress section under results. As shown in Fig. 6c,d,e, all rings displayed a prolonged and controlled release over 181 days, 3.20, 0.94 and 0.96% of estriol (equal 5.32, 16.49 and 22.59 mg) were released from MED-4870 ring with 1% estriol loading, MED-4870 ring with 10% estriol loading, MED-4870 ring with 15% estriol loading silicone rings. The drug–eluting rings provided an average daily release of 0.15, 0.85 and 1.07 mg (from MED-4870 ring with 1% estriol loading, MED-4870 ring with 10% estriol loading and MED-4870 ring with 15% estriol loading, respectively) in the first week, followed by a consistent release of 0.03, 0.09 and 0.13 mg/day/ring which approaches therapeutic concentrations (32). Considering therapeutical dose of estriol, MED-4870 ring with 10% estriol content would be suggested as the optimized formulation. This is in keeping with doses used clinically which is 0.8 mg per week.

## Discussion

We successfully developed a series of medicated pessaries using 3D printed molding with controlled release behaviors of estriol. We found no significant chemical interactions between silicone matrix and adding the estriol. Collectively, our results indicate the feasibility of developing medicated silicone pessary rings with long-term and controlled delivery of estriol through 3D printed molding that could be used to treat patients with pelvic organ prolapse.

The 3D printing technique is a promising platform for fabricating medical scaffolds and devices given its potential to offer customised design and a simple manufacturing process^21^. In this study, we fabricated the pessary rings by using indirect 3D printing to produce the ring molds and loaded them with pre-mixed estriol-silicone materials to overcome low printability and non-uniform mixing problems in the direct 3D printing manufacture. This fabrication method brings the advantage of rapid prototyping and personalised medicines of 3D printing without its extrusion and mixing issues, allowing customised ring dimensions and dose release characters in pessaries. Thus, this study presented a cheap and feasible alternative method of using 3D printed molds to fabricate consistent and accurate pessary rings. Our moulds are generated using the low-cost material nylon. In most instances a generic size estrogen eluting pessary would be used. However, in women where a generic pessary is suboptimal, using 3D printing, we could customise a pessary size and shape. It is envisaged that the clinician would examine the prolapse and obtain measurements of the vagina. These could be used to individualise the size of moulds and customise the pessary.Polymeric systems for the sustained release of therapeutic hormones have been well reported in pharmaceutical formulations^12,22,23^. Silicone monomers have been considered as promising materials for the development of vaginal rings for its durable stability, capability of various drug loadings and slow-release rate of the incorporated medicines. In this study silicone rings with estradiol contents of 1%, 10% and 15% were subsequently fabricated to investigate the feasibility of ring manufacture by 3D printed mold. These pessaries were eluted with estradiol and had similar mechanical properties to commercial pessaries. The MED-4870 silicone ring with 10% drug content released a therapeutic dose of estradiol over 3 months and was therefore considered to be the optimal formulation. After 3 months mechanical properties and estradiol content continued to be robust. There were no interactions seen between the materials used and estradiol release. Due to its denser structure, MED-4870, could provide better drug-holding capability and longer controllable drug release performance. The estradiol-eluting pessary developed in this work released clinically relevant levels of estriol without compromising the mechanical support for pelvic organ prolapse. The rings in the study had similar mechanical properties to commercial pessaries. Due to its denser structure, MED-4870 provided better drug-holding capability and longer controllable drug release performance.

The challenge and problems with ill-fitting non-drug eluting pessaries, could be alleviated by using these medicated pessaries with slow-release estriol. Commercially available non drug eluting pessaries can be suboptimal with regard to comfort and symptoms. Selective Laser Sintering (SLS) 3D printing allows the development of pessary devices in a fraction of the time and cost compared to traditional mold design. This allows us to rapidly prototype devices to meet patient’s needs. Furthermore, eluting our pessary with a slow release estrogen would minimise complications of erosion, vaginal dryness and alleviate difficulty of applying creams vaginally. This will ensure women will not need to use estrogen multiple times a week and will avoid overdosing or underdosing the drug. An estrogen eluting pessary could be used in patients with vaginal atrophy and prolapse to reduce the risk of vaginal erosion and may lead to improved compliance^10^. Importantly, long term low dose estrogen use in women with cystitis is not associated with complications and endometrial monitoring is not required^8,10,24^.

We demonstrated that the mechanical strength of the ring is similar to the mechanical strength of the market sample. Our vaginal pessary releases estradiol over a period of 3 months at a steady state release comprising 30–50 µg/day with a 1% drug content in the ring pessary, a 70–200 µg/day with a 10% drug content in the ring pessary and 100–300 µg/day with a 15% drug content per device. Therefore the 10% drug content may correlate closely with the current therapeutic topical dose of estriol of 0.5–0.8 mg weekly. There are estrogen releasing devices in the form of the E-string which provides estrogen over 3 months. The indication for the E-string is urinary incontinence. The benefit of our estrogen eluting pessary is that one device will assist to deliver estrogen and treat prolapse rather than requiring two devices. Furthermore, the E-string needs to be removed once a pessary is inserted. Therefore, women are often left to choose between the benefits of the E-string versus the pessary. The combined device offers the solution of managing vaginal atrophy and prolapse whilst also offering estrogen treatment commonly used to treat urinary incontinence.Further studies could assess accelerated release to simulate the long-term in vivo release behaviours. A clinical trial is required to evaluate the therapeutic effect of the developed medicated pessary on pelvic organ prolapse.

In this study we developed an estradiol eluting pessary. It had mechanical characteristics similar to commercial pessaries. It released a constant dose of estriol into acidic fluid over a period of 3 months. This estradiol eluting pessary has the potential to become gold standard in treatment of both pelvic organ prolapse and vaginal atrophy.

## Methods

### Preliminary optimization experiments

To evaluate the influence of silicone materials on the drug release rate and mechanical strength, we prepared two silicone films, MED-4830 and MED-4870 and loaded these with different drug concentrations and performed morphological assessment and drug release studies on these samples, as detailed below.

### Preparation of silicone rings

**Figure 7:**
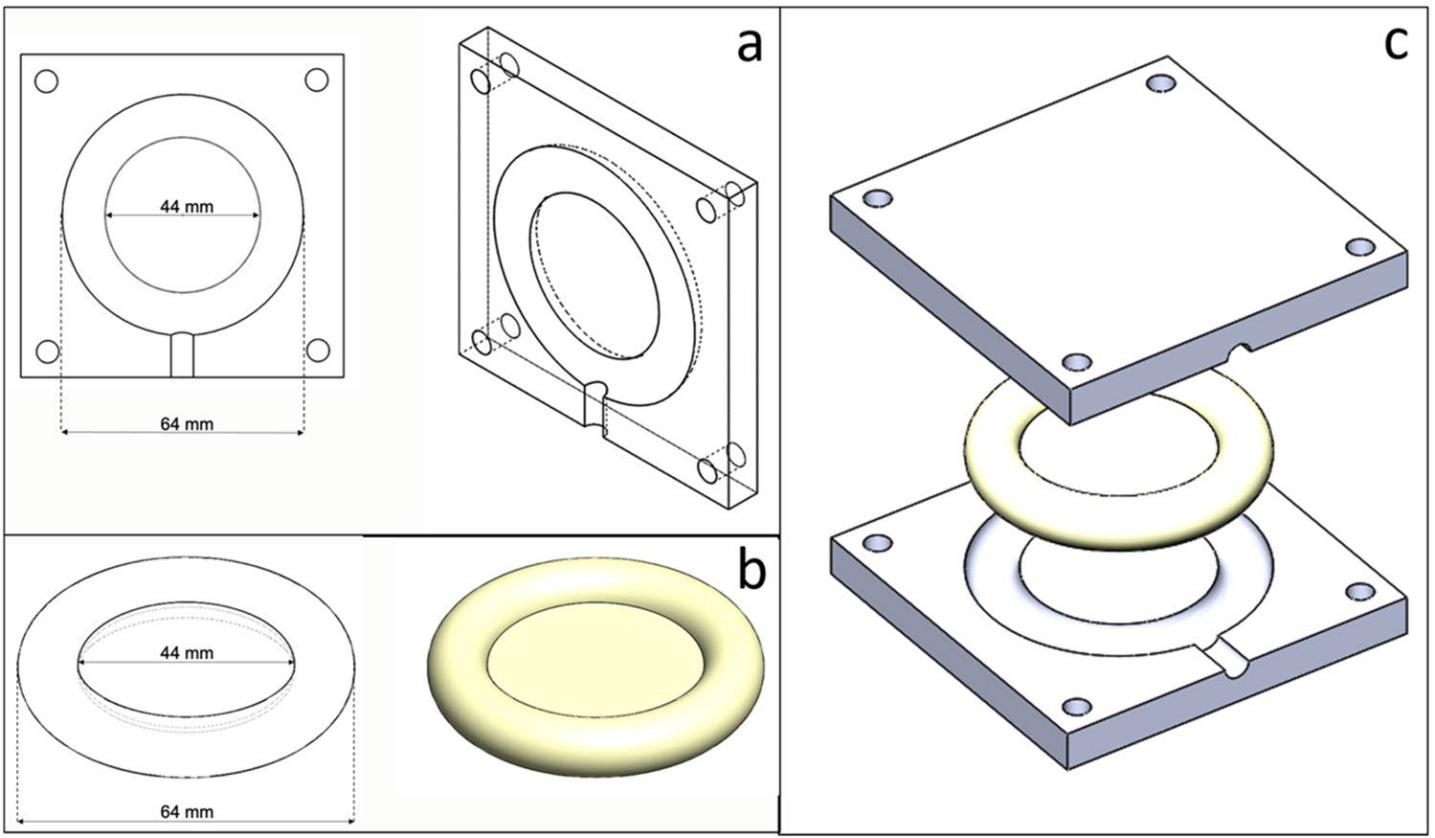
Injection mold and ring design sketch: (**a**) injection mold model, (**b**) ring model and (**c**) mold and ring solid model.

The pessary ring model and the injection mold were designed in SolidWorks computer-aided design (CAD) software (Solidworks 2016 × 64 Edition, Dassault Systemes) and exported as stereolithography (STL) files (Fig. 7). The pessary was designed as a ring with 64 mm outer diameter, which is the most commonly used size, and a central membrane of 1 mm thickness. The injection mold was designed as half-ring shape and manufactured via a Selective Laser Sintering (SLS) 3D printer (Formiga P100, EOS, Germany) with nylon powder (PA2200, EOS, Germany), which can tolerate the heating during silicone curing (160 ℃, 10 min).

Silicone materials, MED-4830 and MED-4870, were kindly gifted by Nusil (Carpinteria, United States). Estriol powder (Flem Pharma, Shanghai, China) at 1%,10% and 15% drug in polymer weight by weight percentage w/w (Table 2) was weighed and gradually added and mixed into the silicon. The silicon in the drug-free pessaries was prepared the same way except estriol powder was not added. The rings were made by filling estriol-mixed or estriol-free silicone paste into two mold halves and fastened with screws followed by heat-introduced curing at 160 °C for 10 min and then stored at room temperature overnight to allow proper curing of the silicone matrix. Afterwards, the samples were removed from the nylon molds and stored at ambient temperature for further characterisation.

**Table 2:** Composition and estradiol content of silicone rings.

	Silicone	PartA:Part B: Estriol (weight)	Drug content (%)*
1	MED-4830	1:1:–	–
2	MED-4830	1:1:0.002	0.1%
3	MED-4830	1:1:0.01	0.5%
4	MED-4830	1:1:0.02	1%
5	MED-4870	1:1:–	–
6	MED-4870	1:1:0.02	0.1%
7	MED-4870	1:1:0.01	0.5%
8	MED-4870	1:1:0.02	1%
9	MED-4870	1:1:0.2	10%
10	MED-4870	1:1:0.3	15%

*Drug content is presented as $\frac{Mass \; of \; drug}{Mass \; of \; silicone}\mathrm{\%}$.

### Morphology assessment

The outer diameter of the silicone rings were measured by an electric Vernier caliper (TD2082, Jaycar Electronics, New Zealand). All parameters were measured at three different positions of each ring and repeated in triplicate samples. The weights of the pessaries were measured using an electronic balance (AUW220D, SHIMADZU, Japan) (n = 3). Cross-section and surface images were obtained employing a Schottky field emission Scanning Electron Microscopy (SEM) (SU-70, Hitachi, United Kingdom) under a working voltage of 5 kilowatt.

### Estriol release from silicone rings

In vitro estriol release from silicone rings (MED-4830 and MED-4870) were studied in simulated vaginal fluids in pH 4.2 (normal vaginal pH) and pH 4.5 (post menopause vaginal pH). Samples were accurately weighed and then placed in a screw top container with 200 mL simulated vaginal fluid. All samples were shaken at 60 rpm at 35 ± 2 °C. At predetermined time intervals, 100 mL of the incubation media from each sample was collected and an equal amount of fresh media was added into each release system to maintain the total volume and sink conditions. The concentration of estriol in the release media was determined by HPLC (see description below). The cumulative amount of estriol released was plotted against time for each sample (n = 3). Estriol-free rings were used as controls. To understand the mechanism of estriol release from silicone rings, kinetics models were used to analyse the release performance (as described in the following section: kinetic models).

The simulated vaginal fluid was prepared with two pH values; pH 4.2 simulating a pre-menopausal vaginal fluid and pH 4.5 to simulate a post-menopausal vaginal fluid, according to a method published previously^25^. Potassium hydroxide (ACS reagent, ≥ 85%), calcium hydroxide (ACS reagent, ≥ 95.0%), urea (ACS reagent, 99.0–100.5%), D-( +)-Glucose (ACS reagent), glycerol (ACS reagent, ≥ 99.5%), lactic acid (meets USP testing specifications), acetic acid (ACS reagent, ≥ 99.7%), and hydrochloric acid (ACS reagent, 37%) were obtained from Sigma-Aldrich (Auckland, New Zealand). Bovine serum albumin (BSA, fatty acid free) was purchased from MP Biomedicals (Auckland, New Zealand). All other used chemicals were analytical grade.

Estriol was measured by high performance liquid chromatography (HPLC) using LC-20AT liquid chromatography LC-20AT HT auto sampler, DGU-20A5 degasser, RF-10A XL UV/VIS detector (Shimadzu USA manufacturing Inc, USA) equipped with a Phenomenex Synergi polar-RP 80A, 4.6 × 250 mm, 4 µm column. A mixture of acetonitrile: 0.1% formic acid in water (50:50) was used as mobile phase at a flow rate of 1 mL/min and injection volume of 10 µL with UV detection at 225 nm.

### Kinetic models

To study the release kinetics and mechanism of estriol release from the silicone film and ring systems, data was fitted to the models shown below:

Zero-order model^26^:

1 $${Q}_{t}={Q}_{0}+{K}_{0}t,$$

where ${Q}_{t}$ is the amount of drug released at time $t$, ${Q}_{0}$ is the initial amount of drug in the solution, and ${K}_{0}$ is the zero-order release constant expressed in the units of concentration/time. Zero-order model describes a system where the release rate of incorporated drug is independent of its concentration^27^.

Higuchi model^26^:

2 $${\mathrm{Q}}_{t}={K}_{H}\times {t}^{1/2},$$

where ${\mathrm{Q}}_{t}$ is amount of drug released in time $t$, ${K}_{H}$ is the Higuchi dissolution constant expressed in the units of concentration/time. Higuchi model is proposed to describe the drug release from a matrix system with different geometrics and porous structure^28^.

### Mechanical test

Compression test of the silicone rings were performed using a texture analyser (TA.XT2, Stable Micro System, Haslemere, Surrey, UK) by compressing the ring for a distance of 10 mm (n ≥ 3 compressions at different sites per ring) and the maximum compression force in each test was recorded^20^. Drug-free and drug-loaded rings were compared to determine whether drug incorporation (and the relative concentration loaded) affects the mechanical property of the rings. Considering the rings are designed for long-term application, the rings treated with SVF for a period of 4-months at 35 ± 2 °C were assessed to evaluate the change of mechanical strength during treatment. A commercial ring (Milex/Cooper Surgical) was used as a reference. Each ring was placed vertically on a ring holder fixed to the platform of the texture analyser. A probe attached to the movable arm was used to compress the ring at a distance of 10 mm at a speed of 2.0 mm/s and the maximum compression forces were recorded.

### Element presence and chemical interactions

In order to identify the drug presence in the silicone matrix and to understand if there is any interactions between estriol and silicone or chemical structure changes in the preparation treatment, spectra obtained from Fourier-transform infrared (FTIR) spectroscopy were used to characterise the presence of specific chemical groups of estriol and silicones and their possible interactions. This was done using a Nicolet iS10 FTIR spectrophotometer (Thermo Scientific, USA). FTIR is used to identify different compounds and their interactions.

### Statistical analysis

A minimum of three technical triplicates were performed for each in vitro study. Data were subjected to one-way analysis of variant (ANOVA) with the level of significance set at p < 0.05.

## Supplementary Information

Supplementary Information.

## Data Availability

Data supporting the findings of this study are available within the paper and Supplementary Materials. Raw data is available from Jingjunjiao Long and Prathima Chowdary upon request.

## References

[1] Swift SE (2000). The distribution of pelvic organ support in a population of female subjects seen for routine gynecologic health care. Am. J. Obstet. Gynecol..

[2] Bai SW (2005). Survey of the characteristics and satisfaction degree of the patients using a pessary. Int. Urogynecol. J..

[3] Sarma S, Ying T, Moore K (2009). Long-term vaginal ring pessary use: discontinuation rates and adverse events. BJOG.

[4] Dessie SG, Armstrong K, Modest AM, Hacker MR, Hota LS (2016). Effect of vaginal estrogen on pessary use. Int. Urogynecol. J..

[5] Vierhout ME (2004). The use of pessaries in vaginal prolapse. Eur. J. Obstet. Gynecol. Reprod. Biol..

[6] de Albuquerque Coelho SC, de Castro EB, Juliato CRT (2016). Female pelvic organ prolapse using pessaries: Systematic review. Int. urogynecol. J..

[7] Wolff B, Williams K, Winkler A, Lind L, Shalom D (2017). Pessary types and discontinuation rates in patients with advanced pelvic organ prolapse. Int. Urogynecol. J..

[8] Mao M (2018). Predictors for unsuccessful pessary fitting in women with symptomatic pelvic organ prolapse: A prospective study. BJOG.

[9] Ma C (2021). Vaginal pessary treatment in women with symptomatic pelvic organ prolapse: A long-term prospective study. Menopause.

[10] Hu JS, Pierre EF (2019). Urinary incontinence in women: Evaluation and management. Am. Fam. Physician.

[11] Wu V, Farrell SA, Baskett TF, Flowerdew G (1997). A simplified protocol for pessary management. Obstet. Gynecol..

[12] Long J (2018). Development of customised 3D printed biodegradable projectile for administrating extended-release contraceptive to wildlife. Int. J. Pharm..

[13] Long J (2019). A 3D printed chitosan-pectin hydrogel wound dressing for lidocaine hydrochloride delivery. Mater. Sci. Eng..

[14] Notelovitz M, Funk S, Nanavati N, Mazzeo M (2002). Estradiol absorption from vaginal tablets in postmenopausal women. Obstet. Gynecol..

[15] Speroff, L. & Group, U. S. V. I (2003). Efficacy and tolerability of a novel estradiol vaginal ring for relief of menopausal symptoms. Obstet. Gynecol..

[16] Collins S, Beigi R, Mellen C, O’Sullivan D, Tulikangas P (2015). The effect of pessaries on the vaginal microenvironment. Am. J. Obstet. Gynecol..

[17] Clemons JL, Aguilar VC, Tillinghast TA, Jackson ND, Myers DL (2004). Patient satisfaction and changes in prolapse and urinary symptoms in women who were fitted successfully with a pessary for pelvic organ prolapse. Am. J. Obstet. Gynecol..

[18] Rose S, Chaudhari A, Peterson CM (2009). Mirena®(Levonorgestrel intrauterine system): A successful novel drug delivery option in contraception. Adv. Drug Deliv. Rev..

[19] Barsky M, Kelley R, Bhora FY, Hardart A (2018). Customized pessary fabrication using three-dimensional printing technology. Obstet. Gynecol..

[20] McCoy CF (2019). Mechanical testing methods for drug-releasing vaginal rings. Int. J. Pharm..

[21] Long J, Gholizadeh H, Lu J, Bunt C, Seyfoddin A (2017). Application of fused deposition modelling (FDM) method of 3D printing in drug delivery. Curr. Pharm. Des..

[22] Cappadoro AJ, Luna JA (2015). Development of an injection molded ethylene-vinyl acetate copolymer (EVA) intravaginal insert for the delivery of progesterone to cattle. Anim. Reprod. Sci..

[23] Manoukian OS (2018). Biodegradable polymeric injectable implants for long-term delivery of contraceptive drugs. J. Appl. Polym. Sci..

[24] Jelovsek JE, Maher C, Barber MD (2007). Pelvic organ prolapse. Lancet.

[25] Murphy DJ (2018). Impact of ring size and drug loading on the pharmacokinetics of a combination dapivirine-darunavir vaginal ring in cynomolgus macaques. Int. J. Pharm..

[26] Dash S, Murthy PN, Nath L, Chowdhury P (2010). Kinetic modeling on drug release from controlled drug delivery systems. Acta Pol. Pharm..

[27] Shaikh HK, Kshirsagar R, Patil S (2015). Mathematical models for drug release characterization: A review. Wjpps.

[28] Higuchi T (1963). Mechanism of sustained-action medication. Theoretical analysis of rate of release of solid drugs dispersed in solid matrices. J. Pharm. Sci..

