# Supplementary material for: An estriol-eluting pessary to treat pelvic organ prolapse
Source: Sci Rep. 2022 Nov 21;12:20021. doi: 10.1038/s41598-022-23791-9 (PMC9681886; doi:10.1038/s41598-022-23791-9)
Supplement: Supplementary file 1 — Supplementary Information. [file 41598_2022_23791_MOESM1_ESM.docx]

# Supplementary:

## FTIR characteristic information:

Pure estriol showed the characteristic bands: 3504 cm^-1^ and 3442 cm^-1^ due to symmetric and asymmetric CH_2_ groups. The vibrations between 2938-2818 cm^-1^ were assigned to C-H stretching in CH_3_, CH_2_ and CH groups. The bands of 1608 cm^-1^ and 1501 cm^-1^ were linked to Skeletal vibrations of the aromatic C-C bonds ^1^.

For characteristic bands of silicone systems, a sharp peak observed at 2964 cm^-1^ was linked to C-H stretching of CH_3_ in silicone monomers. A small peak present at 1413 cm^-1^ was contributed by the CH_3_ asymmetric deformation of Si-CH_3_. The two peaks at 1094 cm^-1^ and 1020 cm^-1^ were assigned for the characteristic band of Si-O-Si asymmetric stretching vibrations ^2^. A small peak at 865 cm^-1^ was present for CH_3_ rocking in Si-CH_3_. The signal of 799cm^-1^ was attributed to Si-O-Si symmetric stretching ^3^.

1 Canbay, H. S., Polat, M. & Doğantürk, M. Study of Stability and Drug-Excipient Compatibility of Estriol. *Bilge International Journal of Science and Technology Research* **3**, 102-107.

2 Park, C.-H. *et al.* Preparation and characterization of (polyurethane/nylon-6) nanofiber/(silicone) film composites via electrospinning and dip-coating. *Fibers and Polymers* **13**, 339-345 (2012).

3 Everaert, E. P., van der Mei, H. C. & Busscher, H. J. Adhesion of yeasts and bacteria to fluoro-alkylsiloxane layers chemisorbed on silicone rubber. *Colloids and Surfaces B: Biointerfaces* **10**, 179-190 (1998).
